# Resting-state cortical electroencephalogram rhythms and network in patients after chronic stroke

**DOI:** 10.1186/s12984-024-01328-7

**Published:** 2024-02-29

**Authors:** Jack Jiaqi Zhang, Zhongfei Bai, Kenneth N. K. Fong

**Affiliations:** 1 Department of Rehabilitation Sciences, The Hong Kong Polytechnic University, Kowloon, Hong Kong SAR China; 2 Department of Rehabilitation, Shanghai YangZhi Rehabilitation Hospital (Shanghai Sunshine Rehabilitation Center), School of Medicine, Tongji University, Shanghai, China

**Keywords:** Stroke, Power spectrum, Connectivity, Brain network, Electroencephalogram

## Abstract

**Objective:**

To investigate the resting-state cortical electroencephalogram (EEG) rhythms and networks in patients with chronic stroke and examine their correlation with motor functions of the hemiplegic upper limb.

**Methods:**

Resting-state EEG data from 22 chronic stroke patients were compared to EEG data from 19 age-matched and 16 younger-age healthy controls. The EEG rhythmic powers and network metrics were analyzed. Upper limb motor functions were evaluated using the Fugl–Meyer assessment-upper extremity scores and action research arm test.

**Results:**

Compared with healthy controls, patients with chronic stroke showed hemispheric asymmetry, with increased low-frequency activity and decreased high-frequency activity. The ipsilesional hemisphere of stroke patients exhibited reduced alpha and low beta band node strength and clustering coefficient compared to the contralesional side. Low beta power and node strength in the delta band correlated with motor functions of the hemiplegic arm.

**Conclusion:**

The stroke-affected hemisphere showed low-frequency oscillations and decreased influence and functional segregation in the brain network. Low beta activity and redistribution of delta band network between hemispheres were correlated with motor functions of hemiplegic upper limb, suggesting a compensatory mechanism involving both hemispheres post-stroke.

## Introduction

The neurophysiological pattern of cortical rhythms can be changed by an acute stroke [1]. Specifically, measured using scalp electroencephalogram (EEG), higher delta power over the bilateral hemispheres correlates with more severe neurological deficits in patients with acute stroke, whereas higher beta power over the bilateral hemispheres correlates with less severe neurological impairment [2]. The ratio of lower-frequency activities in the delta and theta bands to higher-frequency activities in the alpha and beta bands determines the degree of physical disability in patients with acute stroke [3]. Additionally, delta activity over the ipsilesional hemisphere correlates with malignant progression after a large cerebral infarction in the acute stroke stage, whereas beta activity is associated with a benign course [4]. Resting-state EEG power-based measures have been also used as prognostic biomarkers in predicting motor recovery after acute stroke [5]. Longitudinal studies have revealed the predictive value of certain parameters in post-stroke recovery. Specifically, low-frequency oscillations and hemispheric asymmetry measured at baseline showed predictive values for the upper limb motor recovery 6 months after stroke. Saes et al. reported that the hemispheric asymmetry of theta powers is a biomarker that can predict improvements in the Fugl–Meyer assessment-upper extremity (FMA-UE) scores [6]. Additionally, the ratio of lower-frequency activities in the delta and theta bands to higher-frequency activities in the alpha and beta bands was significantly correlated with the level of physical disability, measured using the modified Rankin scale, 6 months post-stroke [7].

In addition to EEG power-based biomarkers, graph theory-based network measures of EEG also show promise to be used as biomarkers in poststroke patients. These measures are computed based on the connectivity matrices among EEG sensors and they can evaluate various aspects of neural networks, such as functional segregation, integration, as well as overall strength of connectivity [8]. Resting-state EEG-based network has been found to be highly correlated with neural networks measured by functional neuroimaging [9]. In the context of poststroke neurophysiology, Shim et al. found that the reduced functional integration of low beta oscillations in chronic stroke patients when moving the affected hand compared to when moving the unaffected hand, suggesting that the EEG-based network property is likely to be relevant to motor functioning in patients after stroke [10].

However, according to a recent review [11], network-based EEG measures are very rarely used in previous studies involving poststroke patients. Most of the studies only evaluated neural reorganization in poststroke brains at the level of connectivity while neglecting the analysis of neural networks [11]. Thus, a systematic investigation using EEG network measures to evaluate functional segregation, integration as well as overall connectivity strength in poststroke population has not yet been carried out.

Despite the advancements in using resting-state EEG biomarkers as diagnostic biomarkers for acute stroke and prognostic indicators for recovery outcome in the subacute stroke phase, there is a significant knowledge gap regarding the neurophysiological alterations and potential biomarkers in patients with chronic stroke. On one hand, it remains largely unknown whether the altered neurophysiological pattern persists in patients with chronic stroke (more than 6 months post-stroke) and correlates with the motor functional levels of the affected upper limb. On the other, network measures based on resting-state EEG have not been well investigated in patients with chronic stroke, and their motor functional correlates remain unknown. Therefore, this study aimed to investigate the persistent neurophysiological patterns after chronic stroke and identify potential biomarkers that correlate with motor functional levels of the upper limb. The specific research objectives included: (1) to examine the stroke-induced hemispheric effect (the ipsilesional *vs.* contralesional hemisphere) on EEG powers and network measures in patients with chronic stroke, (2) to compare the neurophysiological pattern of chronic stroke patients with that of healthy controls, and (3) to study the potential correlation between EEG measures and residual motor functions of the hemiplegic upper limb in patients with chronic stroke.

## Methods

This study recruited 22 individuals after their initial unilateral hemispheric chronic stroke (age: 60.73 ± 7.00 years, 14 men and 8 women; time after stroke onset: 63.73 ± 45.74 months, 13 left hemispheric stroke and 9 right hemispheric stroke, 8 dominant-hand affected, no sign of cognitive impairment, that is, above 6/10 in the abbreviated mental test [12] via convenience sampling). Patients’ hemiparetic arm functions were assessed using the FMA-UE [13] and the action research arm test (ARAT) [14]. Patients’ clinical information related to stroke was collected by reviewing their discharge summaries and/or radiological reports from local hospitals. Table 1 presents patients’ demographic and clinical information. These participants were part of a randomized controlled trial published elsewhere [15]. The current study utilized their resting-state EEG data and analyzed their correlation with clinical scales at baseline (before any intervention was initiated). The human ethics subcommittee of the Hong Kong Polytechnic University approved the study protocol (reference number: HSEARS 20190718003).

**Table 1:** Characteristics of included participants with stroke

Code	Sex	Age (years)	Time after stroke (Months)	Nature of stroke	Lesion location	FMA-UE	ARAT	AMT
1	F	74	71	Ischemic	Subcortical	59	52	10
2	F	61	53	Ischemic	Subcortical	61	54	10
3	M	60	32	Hemorrhagic	Subcortical	22	3	10
4	F	57	206	Ischemic	Unknown	41	20	10
5	M	54	44	Hemorrhagic	Subcortical	43	28	10
6	F	59	59	Hemorrhagic	Subcortical	35	33	10
7	M	73	42	Hemorrhagic	Subcortical	40	11	10
8	F	54	32	Ischemic	Cortical involved	56	30	10
9	M	57	41	Ischemic	Subcortical	35	14	10
10	M	53	29	Ischemic	Cortical involved	40	11	10
11	F	61	31	Hemorrhagic	Cortical involved	52	38	10
12	M	62	134	Ischemic	Cortical involved	27	3	10
13	F	55	16	Hemorrhagic	Subcortical	22	3	10
14	M	62	47	Hemorrhagic	Subcortical	30	6	10
15	M	60	71	Hemorrhagic	Subcortical	61	47	10
16	F	72	139	Ischemic	Subcortical	55	34	10
17	M	44	12	Hemorrhagic	Subcortical	20	3	10
18	M	63	70	Hemorrhagic	Subcortical	57	52	10
19	M	68	45	Ischemic	Subcortical	57	55	10
20	M	65	60	Ischemic	Subcortical	29	8	10
21	M	60	65	Hemorrhagic	Subcortical	55	41	10
22	M	62	103	Ischemic	Cortical involved	39	10	10

*FMA-UE*  Fugl–Meyer Assessment-Upper Extremity, *ARAT*  Action Research Arm Test, *AMT*  Abbreviated Mental Test, *M*  male, *F*  female

In addition, EEG data from 19 age-matched healthy adults (Age: 61.32 ± 11.18, all right-hand dominant, 11 mean and 8 women) and 16 younger-age healthy adults served as the controls (Age: 26.00 ± 1.83 years, all right-hand dominant, eight men and eight women,). We included the data from healthy controls in two different age ranges, in order to rule out any possible age-related neurophysiological alterations apart from those induced by stroke. These healthy individuals participated in other two experimental studies published elsewhere [16] (Reference numbers: HSEARS20180120003; HSEARS20121012008). The participants in this study provided written informed consent before their participation. The resting-state EEG data from patients with stroke and healthy adults at baseline have not been previously published. The current study was a secondary analysis of data generated from previous studies and all these studies were conducted in accordance with the Declaration of Helsinki.

Our EEG data were recorded using a 64-channel cap (Quik-Cap, Compumedics Neuroscan, USA), an EEG Amplifier (SynAmps RT 64-channel Amplifier, Compumedics Neuroscan, USA), and Neuroscan Curry 7 software (Compumedics Neuroscan, USA). Electrode impedance was maintained below 10 kOhm, and the signal was sampled at 1024 Hz. Resting-state EEG was recorded for approximately 3-min with participants seated upright in an electromagnetically shielded EEG chamber with their eyes closed. The participants were instructed to minimize head and body movements during the recording.

The raw EEG signals were downsampled to 250 Hz. Signals with considerable movement artifacts were initially rejected via visual inspection. We filtered the data using band-pass filters within the frequency range of 1–40 Hz. Subsequently, an independent component analysis was utilized to remove any ocular components. The EEG data were referenced to a common average. We defined 26 channels on the left hemisphere (FP1, AF3, F7, F5, F3, F1, FT7, FC5, FC3, FC1, T7, C5, C3, C1, TP7, CP5, CP3, CP1, P7, P5, P3, P1, PO7, PO5, PO3, and O1) and other 26 channels on the right hemisphere (FP2, AF4, F2, F4, F6, F8, FC2, FC4, FC6, FT8, C2, C4, C6, T8, CP2, CP4, CP6, TP8, P2, P4, P6, P8, PO4, PO6, PO8, and O2) to explore the hemispheric effect associated with stroke-induced lesions on cortical EEG rhythms and network features [17]. The data from patients with right-hemispheric stroke were flipped for the convenience of visualization and data analysis.

### Power spectrum analysis

Spectral power values within bins of 0.5 Hz were calculated using Fourier decomposition of data epochs with the *mtmfft* method. The power values within the delta (1–4 Hz), theta (4–8 Hz), alpha (8–12 Hz), beta-1 (12–16 Hz), and beta-2 (16–30 Hz) bands [16] were normalized to the relative percentage of the average power over the five bands for each channel. The calculations were performed using a custom-made MATLAB script modified based on a tutorial of the Fieldtrip toolbox.

(https://www.fieldtriptoolbox.org/workshop/madrid2019/tutorial_stats/).

The hemispheric asymmetry was calculated based on the normalized powers over the ipsilesional and contralesional hemispheres using the following formula [18]:

$$Asymmetric \,\, index= \frac{{Power}_{ipsilesional}-{Power}_{contralesional}}{{Power}_{ipsilesional}{+ Power}_{contralesional}}$$

### Graph-theory-based network analyses

Based on the Hilbert transform, the weighted phase lag index (wPLI) was computed for each frequency band. The wPLI is insensitive to volume conduction, possesses stronger statistical power to detect changes in phase synchronization, and is less affected by uncorrelated noise sources relative to the phase lag index [19]. The wPLI values range from 0 to 1, where a higher value indicates a stronger interregional coupling of neural oscillations and vice versa.

We selected the following three graph theory-based network metrics for this study: (1) node strength, (2) clustering coefficient, and (3) global efficiency. The network metrics were calculated using the brain connectivity toolbox (www.brain-connectivity-toolbox.net) [8]. Density-based thresholding was initially applied to remove spurious connections. The 60-by-60 connectivity matrices were thresholded to retain between 50 to 5% (in 5% increments) of the largest wPLI values [20]. Subsequently, we computed the following network measures for each threshold:

*Node strength* was estimated as the sum of the edge weights connected to the channels.

$${S}_{i}= \sum_{j\ne i}{W}_{ij}$$

where i indicates a specific sensor and j represents the other sensors (j = 1,..., n–1, n = the number of channels). The strength of each channel was computed by summing the strength values of all the connecting channels.

*The Clustering coefficient* was the geometric mean of all triangles associated with each channel, evaluating the functional segregation. First, the values of the neighboring triangles of the channels were calculated using the following:

$${t}_{i}= \frac{1}{2}\sum_{j,h\in N}{({W}_{ij}\times {W}_{jh}\times {W}_{hi})}^\frac{1}{3}$$

where N represents all the channels, and j and h are all possible pairs of neighboring sensors that create triangles with a specific channel. Subsequently, the clustering coefficient for each channel was computed as follows:

$${C}_{i}= \frac{1}{n}\sum_{i\in N}\frac{2{t}_{i}}{{k}_{i}({k}_{i}-1)}$$

where N is the number of sensors and k_i_ is the number of all connected channels for a specific channel.

*Global efficiency* was a measure of functional integration and was calculated as the average of the inverse shortest path length using the following:

$${E}_{global}= \frac{1}{N(N-1)}\sum_{i\ne j}\frac{1}{{l}_{ij}}$$

where l_ij_ is the shortest path length from node j to node i, computed using Dijkstra’s algorithm.

### Statistical analysis

Statistical analyses were performed with SPSS 22.0. Initially, we compared the between-group differences of the hemispheric asymmetry using one-way analysis of variance (ANOVA) with post hoc pairwise Bonferroni-corrected comparisons. Levene’s test for equality of variances was used to test the homogeneity of variances between both groups. When the homogeneity of variance was violated, the degrees of freedom were adjusted using the Welch–Satterthwaite method. Subsequently, we compared the within-hemisphere differences using paired t-tests for the EEG rhythmic powers over different frequency bands, separately for each group. Following previous practice, regional power was defined as the mean power over adjacent electrodes within the following areas: frontal (left side: FP1, AF3, AF7, F1, F3, F5, and F7; right side: FP2, AF4, AF8, F2, F4, F6, and F8), central (left side: FT7, FC5, FC3, FC1, C5, C3, C1, CP5, CP3, CP1, T7, and TP7; right side: FT8, FC2, FC4, FC6, C2, C4, C6, T8, CP2, CP4, CP6, and TP8), and posterior (left side: P7, P5, P3, P1, PO7, PO5, PO3, and O1; right side: P2, P4, P6, P8, PO4, PO6, PO8, and O2) [17]. Finally, the potential correlation between EEG metrics and upper limb clinical scores was explored using Pearson’s correlation in the group of stroke patients. Statistical significance was set at *p* < 0.05 (two-tailed).

Regarding the network outcomes, we initially compared the within-hemisphere differences between the two network measures (node strength and clustering coefficient) under every threshold using paired t-tests for the network measures, separately for each group. Subsequently, the areas under the curve (AUC) were calculated by integrating the measures across the entire threshold range. All 60 channels were included to measure global efficiency, while the channels in the ipsilesional and contralesional hemispheres were computed separately for the other two measurements (i.e., node strength and clustering coefficient). Multiple t tests with Bonferroni corrections were performed to explore the differences in the AUC between the ipsilesional and contralesional hemispheres in patients with stroke, as well as the two control groups. Finally, the potential correlation between the AUC of the network measures and the upper limb clinical outcomes was explored in the group of stroke patients using Pearson’s correlation. Statistical significance was set at *p* < 0.05 (two-tailed).

## Results

### Power analysis

**Fig. 1:**
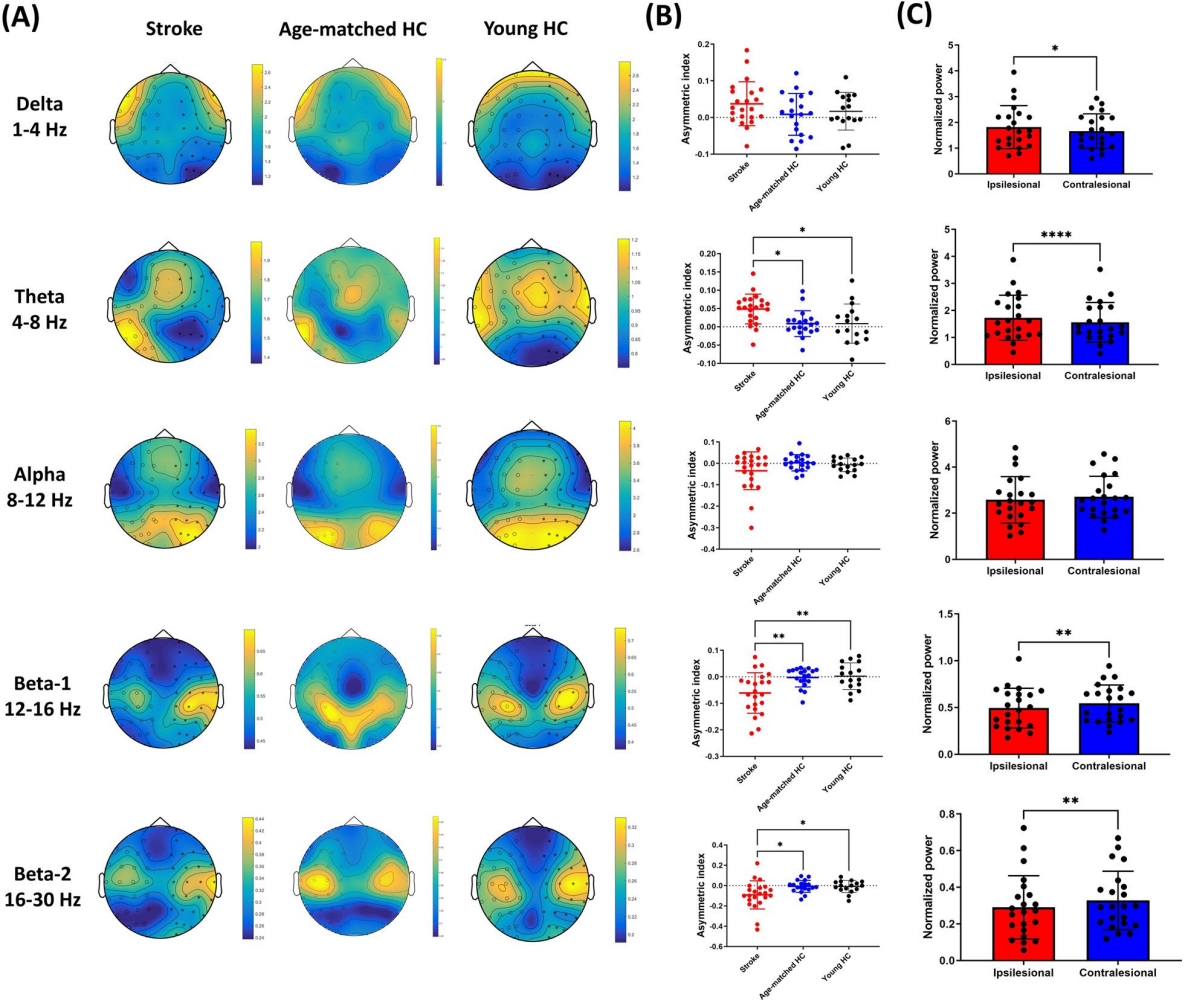
**A** Scalp topographies of electroencephalogram powers over the five frequency bands. Note: The topographies presented in our analysis were derived from the averaged data of participants within each respective group. **B** Comparisons of asymmetry index between the patients with stroke and controls in post hoc pairwise comparisons with Bonferroni corrections. **C** Comparisons of normalized powers between two hemispheres in patients with stroke using paired t tests. *p < 0.05; ** *p* < 0.01; ***p < 0.001, ****p < 0.0001. HC = Healthy controls. Error bars stand for standard deviations

Figure 1A shows the scalp topographies of EEG normalized powers. Significant group effects in hemispheric asymmetry were observed in the theta (F(2,54) = 5.801, *p* = 0.005), beta-1 (F(2,54) = 7.376, *p* = 0.002), and beta-2 (F(2,54) = 4.837, *p* = 0.011) bands. Post hoc pairwise comparisons with Bonferroni corrections were displayed in Fig. 1B, with significant hemispheric asymmetries found in the theta, beta-1 and beta-2 bands in stroke patients in comparison to the two control groups. Paired t-tests revealed within-hemisphere differences in the delta, theta, beta-1, and beta-2 bands in patients with stroke. Compared with the contralesional hemisphere, the ipsilesional hemisphere showed higher power values in the delta (t = 2.33, *p* = 0.030) and theta (t = 4.98, *p* < 0.0001) bands, but lower power values in the beta-1 (t = − 3.16, *p* = 0.005) and beta-2 (t = − 2.92, *p* = 0.008) bands (Fig. 1C). No within-hemisphere differences in cortical rhythmic power were observed in age-matched and young controls.

**Fig. 2:**
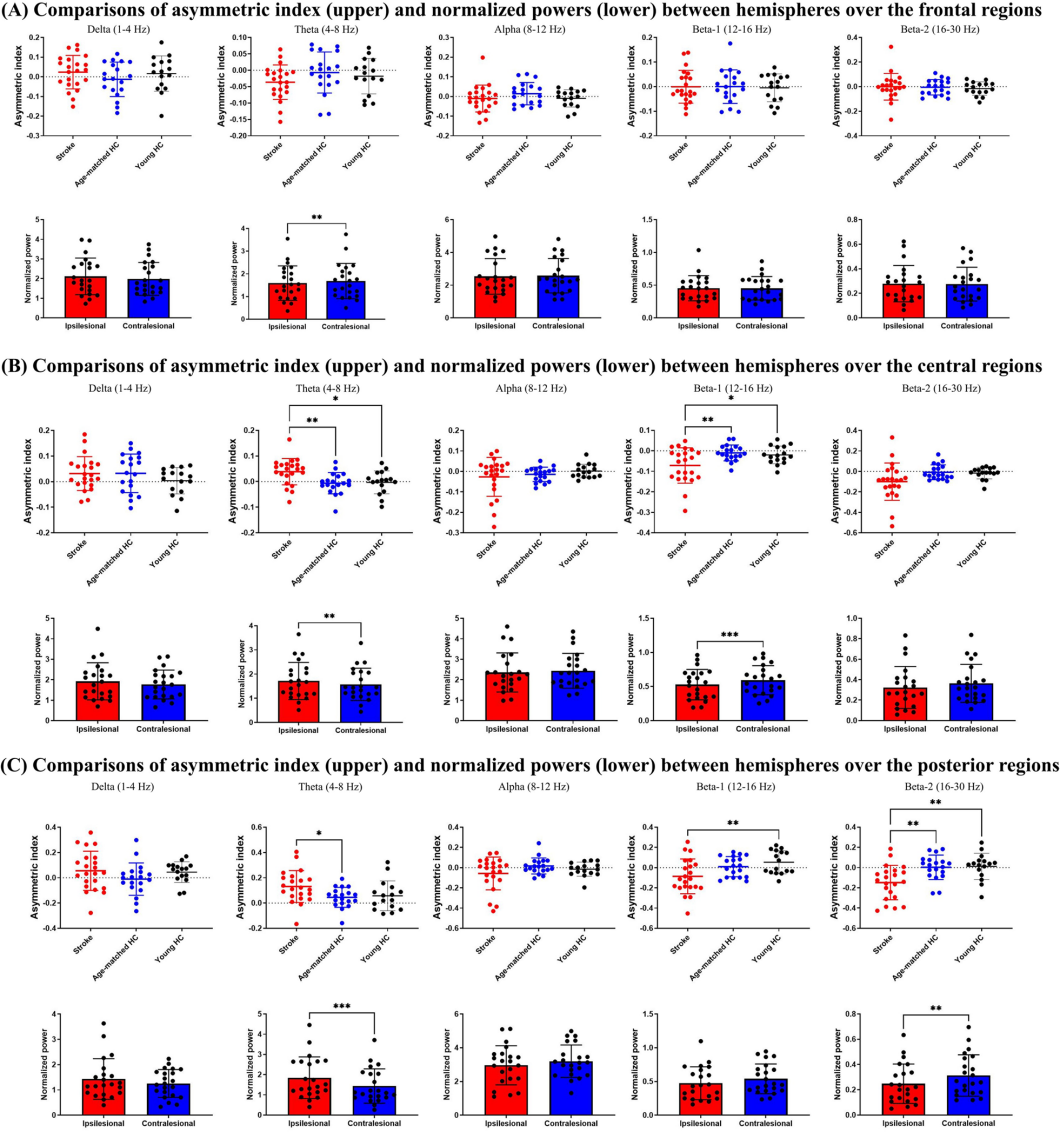
Comparisons of asymmetry index (upper) and normalized powers (lower) over different regions. **A** Frontal, **B** Central, and **C** Posterior regions. The p values in the comparisons of asymmetry index have been corrected using the Bonferroni’s method. *p < 0.05; ** *p* < 0.01. HC = Healthy controls. Error bars stand for standard deviations

When analyzing regional differences, we did not observe any significant group effect in hemispheric asymmetry over the frontal regions. Significant group effects were found in hemispheric asymmetry in the theta (F(2,54) = 5.955, *p* = 0.005) and beta-1 bands (F(2,54) = 5.698, *p* = 0.006) over the central regions, as well as in the theta (F(2,54) = 3.622, *p* = 0.033), beta-1 (F(2,54) = 5.372, *p* = 0.007), and beta-2 (F(2,54) = 7.824, *p* = 0.001) bands over the posterior regions (Fig. 2A). Post hoc pairwise comparisons with Bonferroni corrections were displayed in Fig. 2B. Regarding the within-hemispheric differences, we observed hemispheric differences in the theta band over the frontal regions (t = 2.93, *p* = 0.008, Fig. 2A) and in the theta (t = 3.19, *p* = 0.004) and beta-1 (t = − 3.94, *p* = 0.001) bands over the central regions (Fig. 2B), and in the theta (t = 4.47, *p* < 0.001) and beta-2 (t = − 3.52, *p* = 0.002) bands in the posterior regions (Fig. 2C) of the patients with stroke. The age-matched and young controls had no within-hemispheric differences in cortical rhythmic power over different regions.

A significant correlation was observed between the normalized power in the beta-1 band over the contralesional hemisphere and ARAT scores (r = 0.424, *p* = 0.049). Additionally, a marginally significant correlation was observed between the normalized power in the beta-1 band over the contralesional hemisphere and FMA-UE scores (r = 0.417, *p* = 0.053). Over the central regions, significant correlations were observed between the normalized power in the beta-1 band over the ipsilesional hemisphere and the clinical outcomes (FMA-UE: r = 0.428, *p* = 0.047; ARAT: r = 0.444, *p* = 0.038). Moreover, significant correlations were observed between the normalized power in the beta-1 band over the contralesional hemisphere and clinical outcomes (FMA-UE: r = 0.448, *p* = 0.037; ARAT: r = 0.454, *p* = 0.034).

### Network measures

**Fig. 3:**
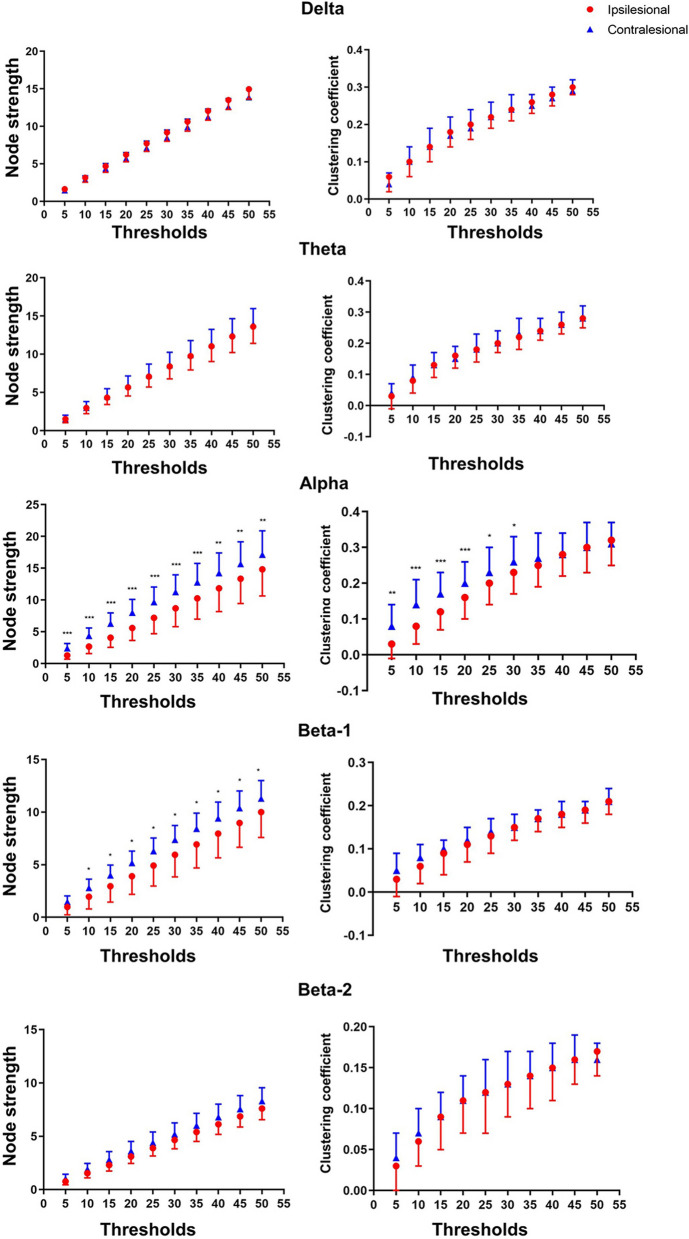
Within-hemisphere comparisons of network measures. Significant within-hemisphere differences were observed in the node strengths at the alpha and beta-1 bands and the clustering coefficient at the alpha band. *p < 0.05; ** *p* < 0.01

Among the patients with stroke, the ipsilesional hemisphere had a significantly lower node strength than the contralesional hemisphere in the alpha and beta-1 bands at all thresholds. The ipsilesional hemisphere had a significantly lower clustering coefficient than the contralesional hemisphere in the alpha band when 30–5% of the largest WPLI values were maintained (Fig. 3). No significant hemispheric differences and within-hemisphere differences in network measures were observed among the age-matched and young controls. As no significant hemispheric differences were observed in healthy controls, their AUCs of the three network measures were computed for all 60 channels, i.e., the global values. A Bonferroni-corrected threshold of significance (*p* = 0.05/6) was applied in comparing the AUCs owing to the six comparisons conducted.

#### Node strength

Patients with stroke demonstrated a reduction of node strength in the alpha band over ipsilesional hemisphere, compared with that of contralesional hemisphere (*p* < 0.001). In addition, the node strength in the beta-2 band over the ipsilesional hemisphere in stroke patients was significantly lower than that in young controls (*p* = 0.002).

Among the correlational analyses with network metrics, we only observed significant correlations between node strengths in the delta band and clinical scales. Specifically, a significant negative correlation was observed between node strengths in the delta band over the ipsilesional hemisphere and clinical scores (FMA-UE: r = − 0.56, *p* = 0.007; ARAT: r = − 0.60, *p* = 0.003). However, a significant positive correlation was observed between the node strength in the delta band over the contralesional hemisphere and clinical scores (FMA-UE: r = 0.47, *p* = 0.026; ARAT: r = 0.65,* p* = 0.021).

#### Clustering coefficients

Both ipsilesional and contralesional hemispheres of stroke patients demonstrated a reduction of clustering coefficient in the beta-1 band, compared with that of young controls (*p* < 0.001).

#### Global efficacy

**Fig. 4:**
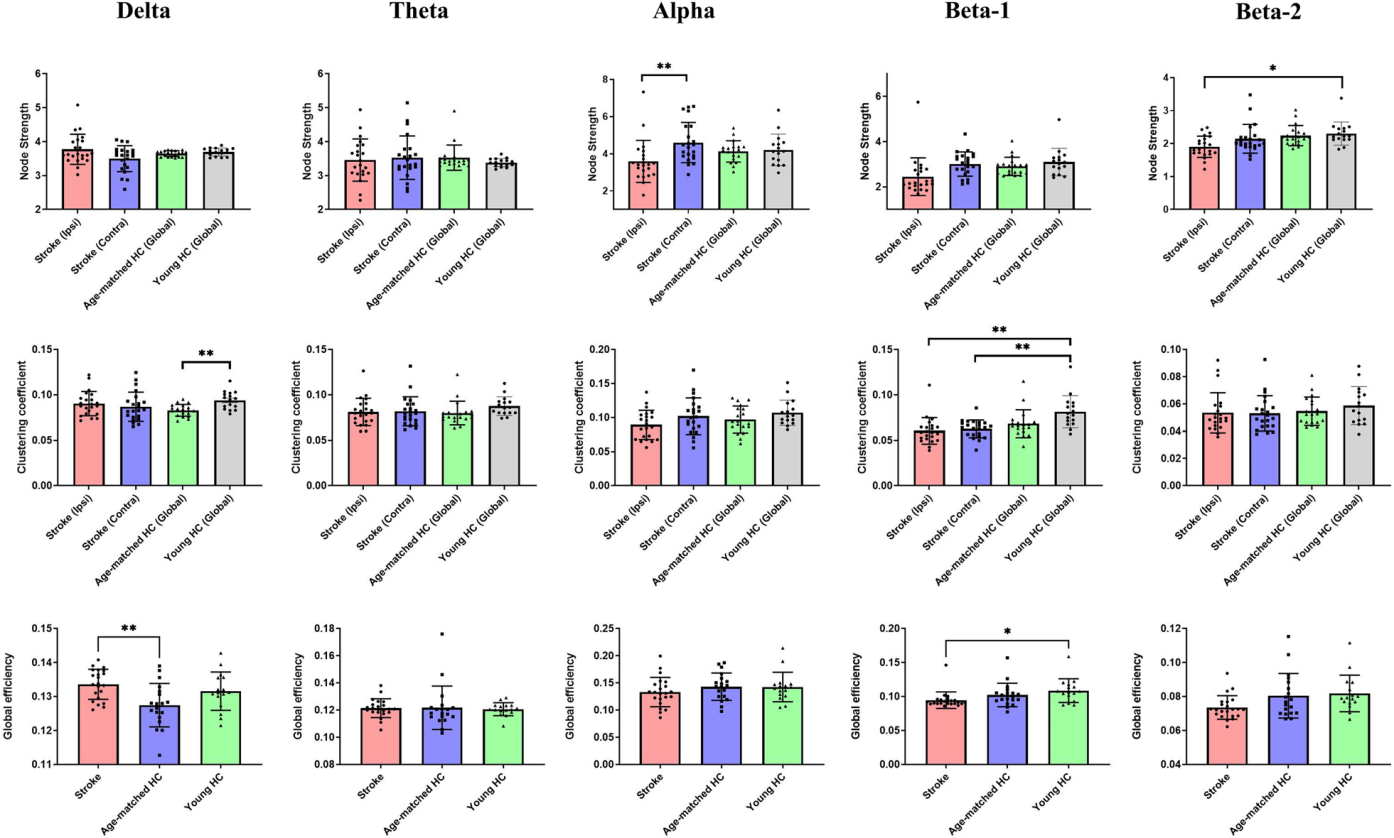
Between-group differences in network measures. The p values in the between-group comparisons of network measures have been corrected using the Bonferroni’s method. *p < 0.05; ** *p* < 0.01. *HC*  Healthy controls. Error bars stand for standard deviations

Significant group effects were found in global efficiency in the theta (F(2,54) = 6.505, *p* = 0.003), beta-1 (F(2,54) = 3.829, *p* = 0.029) and beta-2 bands (F(2,54) = 3.631, *p* = 0.033) among the three groups. Post hoc comparisons were displayed in Fig. 4. Significant differences in global efficiency in the delta band between stroke and age-matched controls, and in the beta-1 band between stroke and young controls were observed, which survived the Bonferroni corrections.

## Discussion

Our study investigated the neurophysiological patterns of cortical rhythmic oscillations in patients with chronic stroke using resting-state EEG measures. Our results revealed that the ipsilesional hemisphere of patients with stroke exhibited increased delta and theta oscillatory activity, while showing a decreased beta oscillatory activity. Hemispheric asymmetry was observed in the theta and beta bands in the patients compared with that in healthy controls. Additionally, the ipsilesional hemispheres of the patients with stroke demonstrated reduced node strength and functional segregation in the alpha and beta bands. These results were robust with the comparisons of two healthy control groups that accounted for age-related effects. However, among the various neurophysiological parameters examined, only the powers at the beta-1 band and the node strength at the delta band over the bilateral hemispheres of the patients correlated with the motor functions of their hemiplegic upper limb after stroke.

Our study revealed asymmetric hemispheric activities in patients with chronic stroke, characterized by a relative increase in slow-rhythmic activities and decrease in fast-rhythmic activities over the ipsilesional hemisphere compared with the contralesional side. This finding is consistent with those of previous studies on patients with acute stroke [1, 21]. However, we did not observe correlation between the level of hemispheric asymmetry and the motor functions of the hemiplegic upper limb. Hemispheric asymmetry has been consistently reported in patients with stroke using different neurophysiological and neuroimaging techniques, leading to the hypothesis of an interhemispheric imbalance model after stroke [22]. However, a recent study using paired-pulse transcranial magnetic stimulation identified no association between interhemispheric asymmetry and motor recovery [23]. Along with this finding, our study suggests that interhemispheric asymmetry could serve as a pathological marker linked to unilateral stroke. However, it does not necessarily indicate a poor recovery outcome at the chronic stage of stroke.

The beta rhythm is associated with activities in the sensorimotor cortex [24]. We observed hemispheric asymmetry of beta activities in patients with stroke; however, the beta-1 activities over the bilateral central regions were correlated with the current functional status, indicating that the ipsilesional and contralesional sensorimotor areas may contribute to motor functions after chronic stroke. This finding suggests a form of adaptive neuroplasticity in bilateral sensorimotor oscillations, where both hemispheres contribute to the motor functions of the paretic upper limb in patients with stroke.

The redistribution of the brain network (the assignment of the new weighting of the bilateral brain networks) may provide new insights into the neurophysiological mechanism of post-stroke motor recovery. The low-frequency oscillation at the delta band is a marker of stroke-related brain injury—a slow delta waveform in the ipsilesional hemisphere of patients with large middle cerebral artery acute ischemic stroke is associated with a malignant progression [4]. Additionally, the power of the delta rhythm is associated with the size of the cerebral infarction in the acute stroke stage [2]. However, we observed no significant correlation between delta power and clinical scales in this study of patients with chronic stroke. These observations indicate that delta oscillation serves as a pathological signature of brain injury resulting from stroke, However, it may not sorely determine the recovery outcome at the chronic phase after stroke.

The delta oscillation also is also supportive to normal brain functioning, including motor outputs [25]. Using network measures, our data revealed that the node strength in the delta band over the ipsilesional hemisphere had an inverse relationship with functional status. In contrast, the node strength in the delta band over the contralesional hemisphere showed a positive relationship with functional status. Due to the contrasting findings observed in the ipsi- and contralesional hemispheres, the correlative results suggest a potential association between the redistribution of brain network in the delta band and the recovery outcome during the chronic stage of stroke. To gain a better understanding of the interhemispheric interaction of hemispheric delta networks following a stroke, further study may utilize dual coils and paired-pulse transcranial magnetic stimulation concurrently in conjunction with EEG recordings. This approach would enable the use of network-based EEG metrics to study the shift of weighting between the ipsilesional and contralesional hemispheric networks in patients with stroke [26].

### Limitations

The current experiment was not free from limitations. First, one major limitation of our study was the unavailability of individual brain imaging data for connectivity and network analyses at the brain source level. However, we used phase synchronization metrics to evaluate functional connectivity at the EEG sensor level, which helped eliminate the volume-conduction challenge when performing connectivity and network analyses. Second, it is worth mentioning that the observed correlations between EEG measures and motor functions were only moderate in magnitude, suggesting that relying solely on EEG biomarkers may not provide a complete understanding of the variation in motor functions among patients after chronic stroke. Future studies should consider investigating the potential synergies between EEG biomarkers and other neurophysiological and neuroimaging biomarkers. A more comprehensive understanding of motor recovery outcomes in poststroke patients may be uncovered via the use of multimodal neural biomarkers. Third, we specifically recruited chronic stroke patients who had experienced a stroke more than one year prior and had reached the recovery plateau. However, in some cases of early chronic stroke, there is a possibility of continued recovery to a limited extent. The small sample size prevents us from drawing conclusions about the potential relationship between time since stroke onset at chronic phase, neurophysiological patterns, and motor functions.

## Conclusion

Patients with chronic stroke showed low-frequency oscillations in the hemisphere affected by the stroke. Interhemispheric asymmetry in the cortical EEG rhythmic power was observed; however, the level of asymmetry was functionally irrelevant. Low beta activity over the bilateral hemispheres was related to the residual arm motor function, indicating a possible compensatory mechanism of motor function after stroke. The hemisphere affected by stroke exhibited reduced influence and functional segregation in the brain network, and the redistribution of the node strength of the brain network in the delta band may serve as a biomarker of motor functioning after stroke.

## Data Availability

The data that support the findings of this study are available on request from the corresponding author, Dr Jack Zhang.
